# New Flavonoid Glycosides from *Elsholtzia rugulosa* Hemsl

**DOI:** 10.3390/molecules14104190

**Published:** 2009-10-20

**Authors:** Gaimei She, Zhiqin Guo, Haining Lv, Dongmei She

**Affiliations:** 1 School of Chinese Pharmacy, Beijing University of Chinese Medicine, Beijing 100102, China; E-Mails: shegaimei@126.com (G.S.); medicine_guo@163.com (Z.G.); seagullning@163.com (H.L.); 2 The Institute of Plant Protection, Chinese Academy of Agricultural Sciences, Beijing 100049, China

**Keywords:** *Elsholtzia rugulos*, flavonoid glycosides, apigenin 4'-*O*-*α*-D-glucopyranoside, 5,7,3',4'-tetrahydroxy-5'-*C*-prenylflavone-7-*O*-*β*-D-glucopyranoside

## Abstract

*Elsholtzia rugulosa* Hemsl. is known in China as a local herbal tea, medicinal herb and honey plant. Chemical examination of *E*. *rugulosa* led to the isolation of two new flavonoid glycosides, apigenin 4'-*O*-*α*-D-glucopyranoside (**1**) and 5,7,3',4'-tetrahydroxy-5'-*C*-prenylflavone-7-*O*-*β*-D-glucopyranoside (**2**), together with nine known flavonoids. Their structures were elucidated on the basis of spectroscopic evidence.

## 1. Introduction

*Elsholtzia rugulosa* Hemsl. (Lamicaeae), which is distributed in the Yunnan, Sichuan and Guizhou provinces of China, is known as a local herbal tea, medicinal herb and honey plant [1]. In these regions, the title plant is also widely used by local people in the treatment of colds, headaches, coughs, pharyngitis and fever [2]. Several flavonoids, maltol glycosides and cyanogenic glycosides have been isolated from *E*. *rugulosa* [3,4]. The antiviral activities of these flavonoids were also reported [4]. As a part of our systematical investigation of Chinese tea and herbal tea plants, and in the search for biologically active flavonoids from plants sources, a detailed study on ethanol extracts of *E*. *rugulosa* was carried out [5,6,7]. This led to the isolation of two new flavonoids glycosides, apigenin 4'-*O*-*α*-D-glucopyranoside (**1**) and 5,7,3',4'-tetrahydroxy-5'-*C*-prenylflavone 7-*O*-*β*-D-glucopyranoside (**2**), together with nine known flavonoids **3**-**11**. Herein, we present the details of this study.

## 2. Results and Discussion

Repeated column chromatography (CC) of the chlorophyll-free fraction of an ethanol extract of *E*. *rugulosa* on Dianion *HP 2MG*L, Sephadex LH-20, MCI-gel CHP-20P, and silica gel, resulted in the isolation of 11 compounds, of which nine known flavonoids were identified as luteolin (**3**) [8] luteolin 7-*O*-*β*-D-glucoside (**4**) [8], luteolin 3'-*O*-*β*-D-glucuronide-6''-methylester (**5**) [9], apigenin (**6**) [10], apigenin 7-*O*-*β*-D-glucoside (**7**) [11], quercetin 3-*O*-*β*-D-glucuronide-6''-methylester (**8**) [12], kaempferol (**9**) [13], 3',4',5,7-tetrahydroxy-8-prenyl-flavone (**10**) [14] and 7,4-dimethylkaempferol (**11**) [15], respectively, by direct comparison with authentic samples or comparison of the spectroscopic data with reported literature values. Among them, compounds **7**-**10** were isolated for the first time from *E*. *rugulosa*. The two new compounds were identified as apigenin 4'-*O*-*α*-D-glucopyranoside (**1**) and 5,7,3',4'-tetrahydroxy-5'-*C*-prenylflavone 7-*O*-*β*-D-glucopyranoside (**2**), and their structures were elucidated as follows.

**Figure 1:**
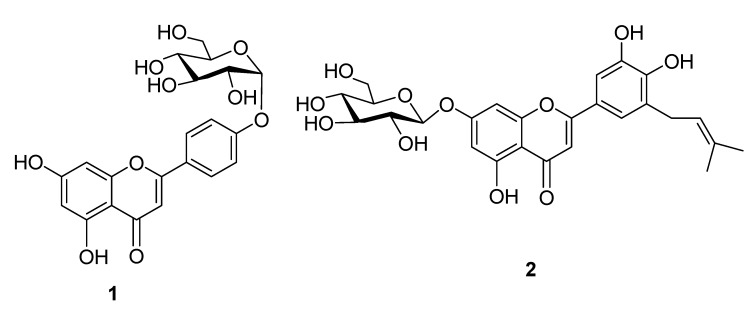
Structures of compounds **1** and **2**.

Compound **1** was obtained as a yellow amorphous powder, and had a molecular formula C_21_H_20_O_10_, derived from its negative HR-FAB-MS (m/z 431.1280 [M-H]^-^) and ^13^C-NMR spectrum. Comparison of the NMR data with those of apigenin [10], and the further 2D-NMR spectral data allow elucidation the structure of compound **1** as shown in Figure 1.

The UV spectrum exhibited absorption maxima at 265 nm (band II) and 331 nm (band I), that are characteristic flavone skeleton bands. The IR spectrum of **1** indicated the presence of hydroxyl (3,376 cm^-1^) and carbonyl functions (1,640 cm^-1^). The occurrence of a flavone skeleton in the molecule could be easily deduced from the ^1^H-NMR spectrum, in which compound **1** showed the signals for an exchangeable proton at δ 12.95 (1H, s), A_2_B_2_-type aromatic protons at δ 7.94 (d, H2', 6') and 6.92 (d, H3', 5') on B-ring, two doublets at δ 6.43 (d, H6) and 6.82 (d, H8) on A-ring, together with an olefinic proton at δ 6.86 (s, H3) on a flavone C-ring. In addition, the ^1^H-NMR also exhibited signals due to one *α*-glucopyranosyl unit [δ 5.42 (d, *J* = 3.7 Hz, H1")]. The *J* value (3.7 Hz) of the anomeric proton indicated the *α*-configuration of the glucose moiety [16]. This was supported by the IR spectrum showing a strong band at 770, 780 cm^-1^, probably due to one glucosyl unit, and the enzymatic hydrolysis displaying the *Rf* values consistent with those of a standard sample of D-glucose, as well as anomeric carbon signal δ 99.9 (C(1')) of *α*-D-glucosyl group observed, in accord with those of literature values [17,18]. The ^13^C-NMR spectrum of **1** exhibited 21 carbons whose aglycon chemical shift were in good agreement with those of apigenin and the sugar moiety chemical shifts were in good agreement with those of *α*-D-glucosyl moiety [18]. The attachment of the glucopyranosyl moiety was deduced to be at C-4′ according to glycosylation rule. The conclusion was further confirmed by the HMBC spectrum in which the anomeric proton of the glucopyranosyl moiety at δ 5.42 (d, H1'') showed long range correlation with C(4') (δ 161.1). Therefore, the structure of **1** was determined to be apigenin 4'-*O*-*α*-D-glucopyranoside.

Compound **2** was obtained as a pale yellow amorphous powder. The molecular formula C_26_H_28_O_11_ was derived by negative ion HR-FAB-MS (m/z: 515.1913 [M-H]^-^) in combination with the presence of 26 carbon signals in its ^13^C-NMR spectrum, and the further 2D-NMR spectral data allow to elucidate the structure of compound **2** as shown in Figure 1.

**Figure 2:**
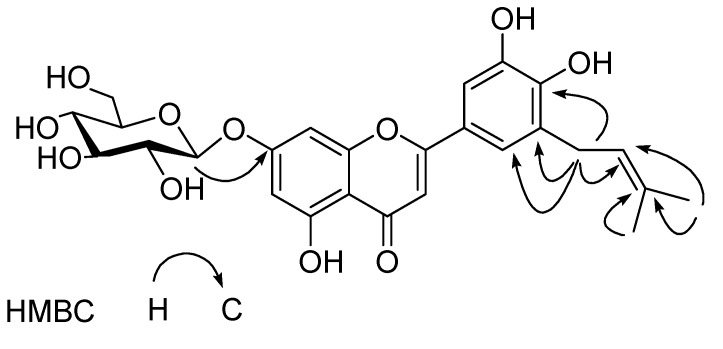
Key HMBC correlations of **2**.

The signals at δ 6.78 (s, H3) ascribable to C_3_- proton on a flavone C-ring, and two aromatic proton signals at δ 6.61 (d, H6) and 6.48 (d, H8) due to H-6, 8 on A-ring protons, respectively, two broad singlet signals at high field in the aromatic region [δ 7.40 (d, H2'), 6.95 (d, H6')] on B-ring were observed in ^1^H-NMR spectrum, which suggested the occurrence in the molecule of a flavone skeleton with a tetra-substituted B-ring. In addition, one glucopyranosyl unit [δ(H) 5.08 (d, H1"), δ(C) 101.9 C(1'')] was evident in the ^1^H- and ^13^ C-NMR of **2**. On enzymatic hydrolysis, compound **2** liberated D-glucose and the *J* value (8.1 Hz) of the anomeric proton concluded the *β*-configuration of the D-glucose moiety. The HMBC correlations of glucosyl H-1'' [δ 5.08 (d, H1'')] in **2** with the C(7) (δ 166.7) confirmed that the location of glucopyranosyl groups were at C-7 in **2**. These NMR features were resembled to those of luteolin 7-*O*-*β*-D-glucoside (**4**) [8], except for the existence of an additional set of signals arising from a prenyl group in **2**. Characteristic signals of prenyl group were observed at δ 1.68 (s, H5'''), 1.62 (s, H4'''), 3.62 (m, H1'''), 5.16 (br. t, H2'''), confirming that **2** was a prenylated flavone glycoside [20]. The downfield chemical shift of C(5') of **1** at δ 127.1 indicated that the additional prenyl group was linked at the C(5') position, which was further confirmed by the HMBC correlations of H1''' (δ 3.62) of prenyl unit with the carbon at δ 127.1 C(5')) of the flavone glycoside (Figure 2). On the basis of the above evidence, the structure of **2** was elucidated as 5,7,3',4'–tetrahydroxy-5'-*C*-prenylflavone 7-*O*-*β*-D- glucopyranoside.

## 3. Experimental

### 3.1. General

Column chromatography (CC) was performed on Dianion *HP 2MG*L (Mitsuishi Chemical Co.), Sephadex LH-20 (Pharmacia Fine Chemical Co. Ltd.), MCI-gel CHP20P (Mitsubishi Chemical Co.) and silica gel (Qingdao Haiyang Chemical Co.). TLC was carried on silica gel G precoated plates (Qingdao Haiyang Chemical Co.) with CHCl_3_-MeOH-H_2_O (9:1:0.1 or 7:3:0.5). The spots were detected by spraying with 10% H_2_SO_4_ ethanol solution, followed by heating. UV spectra were obtained on a UV 210A Shimadzu spectrometer (Shimadzu, Kyoto, Japan). IR spectra were recorded on a Shimadzu IR-450 spectrometer as KBr pellets. ^1^H- and ^13^C-NMR, HSQC and HMBC spectra were recorded with Bruker AM-400 and DRX-500 spectrometers operating at 500 and 400 MHz for ^1^H, and 125 and 100 MHz for ^13^C, respectively. FABMS and HRFABMS were recorded on an AutoSpec 3000 spectrometer (VG, Manchester, UK) with glycerol as the matrix.

### 3.2. Plant Material

The aerial parts of *E*. *rugulosa* were collected from Yunnan Province, China. The voucher specimen (No. 0215159) was deposited in the KUN Herbarium of Kunming Institute of Botany, Chinese Academy of Sciences.

### 3.3. Extraction and Isolation

Dried plant material (400 g) of *E*. *rugulosa* was refluxed four times with ethanol (4.0 L) for 3 h. After removal of the organic solvent under reduced pressure, the aqueous solution afforded precipitates, which were removed by filtration, and the filtrate was partitioned with ethyl ether to yield ethyl ether and aqueous fractions. The aqueous fraction was concentrated to a small volume (120 mL) and applied to a Dianion *HP 2MG*L column, eluting with H_2_O-MeOH (1:0-0:1) to afford five fractions (fr. 1-5). Fr. 2 (0.4 g) was subjected to CC on silica gel (CHCl_3_-MeOH-H_2_O, 9:1:0.1-7:3:0.5), Sephadex LH-20 and MCI-gel CHP-20P, eluting with H_2_O-MeOH (1:0-0:1) to afford compounds **2** (6 mg) and **5** (15 mg). Fr. 3 (1.7 g) was subjected to CC on silica gel (CHCl_3_-MeOH-H_2_O, 9:1:0.1-7:3:0.5), Sephadex LH-20 (H_2_O-MeOH, 1:0-0:1) and MCI-gel CHP-20P (H_2_O-MeOH, 1:0-0:1) to afford **1** (8 mg), **3** (34 mg), **4** (59 mg) and **11** (16 mg). Repeated CC on Sephadex LH-20 and MCI-gel CHP-20P, eluting with H_2_O-MeOH (1:0-0:1), respectively, gave **7** (9 mg), **8** (10 mg) and **9** (50 mg) from Fr. 4 (1.7 g), and **6** (22 mg) and **10** (24 mg) from Fr. 5 (0.7 g).

*Compound* **1**: Yellow amorphous powder. UV-Visible λ_max_ (nm) MeOH: 265, 296, 331; IR (KBr, cm^-1^): 3.376, 1.640, 1.613, 1.508, 1.055, 780, 770; ^1^H NMR (500 MHz, DMSO-d_6_): 7.94 (d, *J* = 8.6 Hz, H2', H6'), 6.92 (d, *J* = 8.6 Hz, H3', H5'), 6.86 (s, H3), 6.82 (d, *J* = 1.6 Hz, H8), 6.43 (d, *J* = 1.6 Hz, H6), 5.42 (d, *J* = 3.7 Hz, H1''), 5.10 (d, *J* = 3.9, 12.5 Hz, Ha6''), 5.05 (dd, *J* = 3.9, 12.5 Hz, Hb6''), 4.63 (m, H2''), 3.71 (m, H4''), 3.17-3.58 (m, H3'', H5'') ppm; ^13^C-NMR (125 MHz, DMSO-d_6_): 181.9 (s, C-4), 162.9 (s, C-2), 161.4 (s, C-5), 161.1 (s, C-4'), 156.9 (s, C-9), 128.6 (d, C-2', C-6'), 121.0 (s, C-1'), 116.0 (d, C-3', 5'), 105.4 (s, C-10), 103.1 (d, C-3), 99.9 (d, C-1''), 99.5 (d, C-6), 94.8 (d, C-8), 77.2 (d, C-3''), 76.3 (d, C-5''), 73.1 (d, C-2''), 69.5 (d, C-4''), 60.6 (t, C-6''); HR-FAB-MS (neg.): 431.1280 [M-H]^-^ (calcd. for C_21_H_19_O_10_ 431.1102).

*Compound* **2**: Yellow amorphous powder. UV-Visible λ_max_ (nm) MeOH: 256, 267, 346; IR (KBr, cm^-1^): 3,450, 2,920, 1,650, 1,573, 1,515, 990-600; ^1^H-NMR (500 MHz, MeOH+DMSO-d_6_): 7.40 (d, *J* = 1.9 Hz, H2'), 6.95 (d, *J* = 1.9 Hz, H6'), 6.78 (s, H3), 6.61 (d, *J* = 1.8 Hz, H6), 6.48 (d, *J* = 1.8 Hz, H8), 5.16 (br t, *J* = 6.7 Hz, H2'''), 5.08 (d, *J* = 8.1 Hz, H1''), 3.78-4.40 (m, H2'', H3'', H4'', H5''), 3.62 (m, H1'''), 1.68 (s, H5'''), 1.62 (s, H4''') ppm; ^13^C-NMR (125 MHz, MeOH+DMSO-d_6_): 183.7 (s, C-4), 166.7 (s, C-7), 162.1 (s, C-5), 158.9 (s, C-9), 151.1 (s, C-4'), 147.3 (s, C-3'), 130.1 (s, C-3'''), 123.7 (s, C-1'), 127.1 (s, C-5'), 122.1 (s, C-2'''), 117.6 (d, C-6'), 114.6 (d, C-2'), 107.1 (s, C-10), 104.5 (s, C-3), 101.9 (d, C-1''), 101.0 (d, C-6), 96.1 (d, C-8), 78.5 (d, C-3''), 77.9 (d, C-5''), 74.7 (d, C-2''), 71.2 (d, C-4''), 62.9 (t, C-6''), 28.4 (t, C-1'''), 25.6 (q, C-5'''), 17.8 (q, C-4'''); HR-FAB-MS (neg.): 515.1913 [M-H]^-^ (calcd for C_26_H_27_O_11_ 515. 1657).

### 3.4. Enzymatic hydrolysis of compounds ***1*** and ***2***

An aqueous solution of **1** (3 mg) and maltase (1 mg) was incubated at 37 °C for 80 h. The solution was extracted with CHCl_3_ and aglycone produced was identified as apigenin by comparison with compound **6** on silica gel TLC using CHCl_3_-MeOH-H_2_O (8:2:0.2), *R_f_* = 0.68. The aqueous layer was concentrated to a residue, which was dissolved by water and examined for identification of the component sugar, and D-glucose was identified by direct comparison on silica gel TLC with an authentic sample, using CHCl_3_-MeOH-H_2_O (7:3:0.5). *R_f_* = 0.23.

A solution of **2** (2 mg) in H_2_O (1 mL) were treated with crude cellulase (7 mg) at 37 °C for 60 h. The reaction mixture was diluted with H_2_O (2 mL), and extracted with CHCl_3_ (3 mL × 2). The aqueous layer was concentrated to a residue, which was dissolved by water and examined for identification of the component sugar, and D-glucose was identified by direct comparison on silica gel TLC with an authentic sample, using CHCl_3_-MeOH-H_2_O (7:3:0.5). *R_f_* = 0.23.

## 4. Conclusions

A detailed phytochemical investigation on *E*. *rugulosa* led to the isolation of two new flavonoid glycosides, apigenin 4'-*O*-*α*-D-glucopyranoside (**1**) and 5,7,3',4'-tetrahydroxy-5'-*C*-prenylflavone-7-*O*-*β*-D-glucopyranoside (**2**), together with nine known compounds (**3**-**11**). Among them, compounds **7**-**10** were isolated for the first time from *E*. *rugulosa*.

## References

[1] Wu C.Y. (1988). Flora of China.

[2] Jiangshu New College of Medicine (1985). The Dictionary of Chinese Medicine.

[3] Liu A.L., Liu B., Qin H.L., Lee S.M., Wang Y.T., Du G.H. (2008). Anti-influenza virus activities of flavonoids from the medicinal plant *Elsholtzia rugulosa*. Planta Med..

[4] Li H.Z., Nakashima T., Tanaka T., Zhang Y.J., Yang C.R. (2008). Two new maltol glycosides and cyanogenic glycosides from *Elsholtzia rugulosa* Hemsl. J. Nat. Med..

[5] He Z.D., Liu Y.Q., Yang C.R. (1992). Glycosides from *Ligustrum purpurascens*. Acta Bot. Yunnanica.

[6] Ouyang M.A., Wang H.Q., Liu Y.Q., Yang C.R. (1997). Triterpenoid saponins from the leaves of *Ilex latifolia*. Phytochemistry.

[7] She G.M., Wang D., Zeng S.F., Zhang Y.J., Chang C.R. (2008). New antioxidative phenylethanoids and sugar esters from Ku-Ding tea (the leaves of *Ligustrum purpurascens*). J. Food Sci..

[8] Chen H.Y., Zhou C.X., Lou Y.J., Duan Z.H., Zhao Y. (2005). Chemical constituents from *Elsholtzia blanda*. Zhongguo Zhong Yao Za Zhi.

[9] Ma J.Y., Wang Z.T., Xu L.S., Xu G.J. (1999). A sesquiterpene lactone glucoside from *Ixeris denticulata* f. pinnatipartita. Phytochemistry.

[10] Shen C.C., Chang Y.S., Ho L.K. (1993). Nuclear magnetic resonance studies of 5, 7-dihydroxy flavonoids. Phytochemistry.

[11] Jiang L., Yao Q.Q., Xie Y.Y. (2009). Study on chemical constituents of *Sonchus arvensis* L. Food Drug.

[12] Zhang R.L., Sun X.C., Li W.X., Wu L.J., Huang J., Sun B.H. (2008). Isolation and identification of chemical constituents of *Polygonum perfoliatum* L. J. Shenyang Pharm. Univ..

[13] Zhou Z.H., Yang C.R. (2000). Chemical constituents of crude green tea, the material of Pu-er tea in Yunnan. Acta Bot.Yunnanica.

[14] Yang L., Che Q.M., Bi C., Sun Q.S. (2007). Flavonoid compounds in solid wastes of *Radix Glycyrrhizae*. Chin. Tradit. Herb. Drugs..

[15] Zhao Y., Lin Q.C., Zhao Y., Chen Y.G. (2004). Studies on the constituents from the herb of *Elshotzia rugulosa*. Zhongguo Zhong Yao Za Zhi.

[16] Wang X.K. (1988). Natural Medicinal Chemistry.

[17] Mathela D.K., Pant A.K., Mathela C.S. (1984). A pyrone glycoside from *Erigeron karwinskyanus*. Phytochemistry.

[18] Gao Y.M., Wang M.Z., Wang J.P., Zhao Q., Qin H.Y., Mu H J., Guan G.J. (1995). Chemical constituents from *Lonicera japonica*. Chin. Tradit. Herb. Drugs..

[19] Markham K.R., Ternai B., Stanly R., Geiger H., Mabry T.J. (1978). ^13^ C-NMR studies of flavonoids-III. Tetrahedron.

[20] Bohlmann F., Abraham W.R. (1979). Neus Prenylflavone aus *Helichrysum hypocephalum*. Phytochemistry.

